# Study on upper bound limit analysis of horizontal layers slope stability based on optimization method

**DOI:** 10.1038/s41598-023-33373-y

**Published:** 2023-04-13

**Authors:** Yong Yao, Zhen Wang

**Affiliations:** 1 School of Mining, Liaoning Technical University, Fuxin, Liaoning China; 2 School of Engineering, Huanghe Science and Technology College, Zhengzhou, Henan China

**Keywords:** Natural hazards, Engineering

## Abstract

Slope stability is an important problem in the field of geotechnical engineering. In order to widen the application range of upper bound limit analysis in engineering practice, this paper analyzes layered distribution characteristics of slope soil and establishes horizontal layers slope failure mechanism which satisfies the separation of velocity, then proposes calculation method for external force power and internal energy dissipation power using discrete algorithm. On this basic, this paper prepares cycle flow of slope stability analysis by upper bound limit principle and strength reduction principle, and develops stability analysis system by computer programming technology. Taking typical mine excavation slope as engineering background and calculates stability coefficient corresponding to different slope angles and evaluate analysis results accuracy by combining with limit equilibrium method. The results show that the stability coefficient error rate of two methods is between 3 and 5%, which can satisfy requirements of engineering practice. Moreover, stability coefficient obtained by upper bound limit analysis is an upper limit solution, and calculation errors are easy to eliminate, so it has certain applicability in slope engineering practice.

## Introduction

Slope stability is an important problem in geotechnical engineering, it is widely involved in highway, railway, water conservancy, construction, mining and other fields^1,2^. Nowadays, commonly used stability analysis methods mainly include limit equilibrium method, limit analysis method and so on^3–5^.

Limit equilibrium method divides the soil in potential sliding surface into several blocks, and calculates stability coefficient by establishing static equilibrium equation and torque equilibrium equation of each block and whole sliding surface based on Mohr–Coulomb yield criterion^6–10^. Limit equilibrium method contains clear mechanical theory, strong computational convergence and widely engineering applications^11^. However, it is generally believed that the number of unknown conditions is greater than the number of equilibrium equations when limit equilibrium method is used for stability analysis^12^. It is necessary to introduce some putative conditions to ensure the problem can be solved due to the established equilibrium equations belong to a hyperstatic problem. According to the difference between putative conditions, limit equilibrium method forms simplified Bishop method, simplified Janbu method, Morgenstern–Price method, Sarma method, Spencer method and so on^13–17^. In most of limit equilibrium methods, scholars determined distribution state of normal stress through hypothesis inter-bar force. Although the calculation process can be simplified, the calculated normal stress distribution state is different from actual stress state, which will lead to a certain deviation in calculation results inevitably^18–20^. Moreover, limit equilibrium method analyzes slope stability from angle of static equilibrium and torque equilibrium, and this method only can calculate slope stability coefficient and the most dangerous sliding surface without considering stress–strain relationship of slope soil effectively^21^. As a result, this method can’t reflect influence of stress loading path, stress loading history and other factors on slope stability^22^. Therefore, it is generally considered that limit equilibrium method is only an approximate solution in slope stability analysis, rather than a rigorous calculation method^23^. Aim at shortcomings of limit equilibrium method, limit analysis method only calculates limit load values of slope soil when it enters fluid plastic stage, this method fully considers stress–strain relationship of soil materials, and it is suitable for slope stability analysis of various mechanical characteristics^24^. Limit analysis method includes upper bound limit analysis and lower bound limit analysis^25^. Among them, upper bound limit analysis is based on upper bound limit principle and establishes movement allowable velocity field by deformation coordination condition. Meanwhile, upper bound limit analysis obtains upper limit of actual load values combined with functional analysis method^26,27^. Lower bound limit analysis is based on lower bound limit principle and establishes static allowable stress field by boundary yield condition. Meanwhile, lower bound limit analysis obtains lower limit of actual load values combined with functional analysis method^28,29^. The actual load of slope soil can be defined in a reasonable range by upper bound limit analysis lower bound limit analysis^30^. Comparing with two methods, establishment of movement allowable velocity field is more convenient to realize, so upper bound limit analysis has a wider range of application in engineering practice^31^. Research on upper bound limit analysis began in the mid-twentieth century, Drucker established flow law associated with yield criterion on the premise of stable materials. Based on static allowable stress field and movement allowable velocity field of stable materials, limit analysis method was established and applied to solve problems of limit load^32^. Chen applied upper bound limit analysis to the field of geotechnical engineering, and introduced the application of this method in slope stability analysis^33^. Michalowski established slope failure mechanism which meet requirements of velocity separation, and proposed stability analysis method based on limit equilibrium conditions, which effectively solved kinematic problems in slope failure^34^. After nearly a century development, upper bound limit analysis has developed from a simple empirical algorithm to an important analysis method with a complete theoretical system, and is widely used in engineering practice^23^. What is more, upper bound limit analysis also has its prominent disadvantages. Due to restrictions of solving process in plastic analysis, upper bound limit analysis provides a perfect solution for homogeneous slopes with simple form, but applicability of this method is greatly restricted for slope stability analysis under complex geological conditions^35^.

Obviously, upper bound limit analysis has its unique advantages. But the applicability of this method is limited due to characteristics of horizontal layered distribution of soil in engineering practice. Therefore, this paper establishes slope failure mechanism based on layered distribution characteristics analysis of slope soil and proposes calculation method for external force power and internal energy dissipation power using discrete algorithm. What is more, develops stability analysis system by computer programming technology and applies the analysis system into slope engineering practice, and evaluate analysis results accuracy by combining with simplified Bishop method. The results of this paper can solve the problem of horizontal layers slope stability analysis and broaden the application range of upper bound limit analysis, so it has important theoretical significances and engineering values.

## Calculation method of upper bound limit analysis for slope stability

### Establishment of computational model

**Figure 1:**
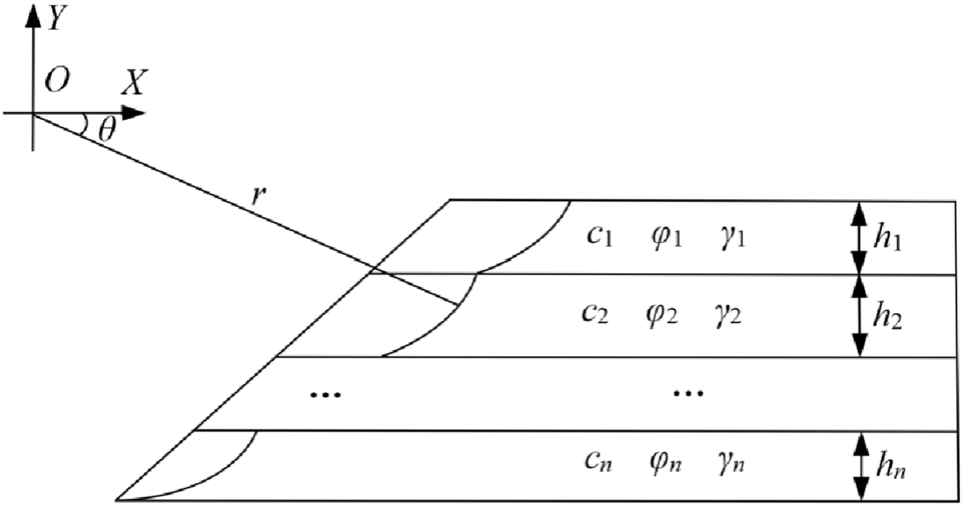
Failure mechanism of horizontal stratified slope.

According to associated flow law and velocity separation requirements, the failure mechanism of homogeneous slope is logarithmic spiral when shear failure occurs^36^. For horizontal layered slope, it can be approximated as superposition of homogeneous slopes in longitudinal direction. Therefore, for horizontal layered slope composed of soil mass in *n* layers, the failure mechanism is formed by the combination of *n* logarithmic spirals. The failure mechanism of horizontal stratified slope is shown in Fig. 1.

For horizontal layered slope composed of soil mass in *n* layers, the slope angle is *α*, then the side slope equation can be expressed as *x* = *f*(*y*) = cot*α*·*y* + *b*. What is more, morphological characteristics and mechanical parameters of soil in each layer are defined as follows. The thickness of each layer soil is *h*_1_, *h*_2_, *h*_3_…*h*_*n*_, the density of each layer soil is *γ*_1_, *γ*_2_, *γ*_3_…*γ*_*n*_, the cohesion of each layer soil is *c*_1_, *c*_2_, *c*_3_…*c*_*n*_, the internal friction angle of each layer soil is *φ*_1_, *φ*_2_, *φ*_3_…*φ*_*n.*_ Suppose equation of slope bottom line is *y* = *y*_0_, then the roof equation of the *m*th layer soil (m = 1, 2, 3…*n*) can be expressed as *y* = *y*_0_ + *h*_*n*_ + *h*_*n*−1_⋯ + *h*_*m*_ and the bottom equation of the *m*th layer soil can be expressed as *y* = *y*_0_ + *h*_*n*_ + *h*_*n*−1_⋯ + *h*_*m*+1_ based on the thickness of each layer soil. At the same time, according to the unique velocity of failure mechanism at soil interfaces, it can be determined that failure mechanism in each soil layer has same angular velocity *ω* and rotation center *O*. The logarithmic spiral equation of the *m*th layer soil can be expressed as

1 $$ r_{m} (\theta ) = a_{m} \cdot e^{{\theta \cdot \tan \phi_{m} }} . $$

In Formula (1), *a*_*m*_ is an undetermined parameter of logarithmic spiral equation for the *m*th layer soil, *a*_*m*_ > 0. It can obtain Formula (2) according to convert Formula (1) to plane rectangular coordinates.

2 $$ \left\{ \begin{gathered} \frac{{x_{m} }}{{a_{m} }} = e^{{\theta \cdot \tan \phi_{m} }} \cdot \cos \theta \hfill \\ \frac{{y_{m} }}{{a_{m} }} = - e^{{\theta \cdot \tan \phi_{m} }} \cdot {\text{sin}}\theta \hfill \\ \end{gathered} \right.. $$

Formula (2) is logarithmic spiral equation of the *m*th layer soil in plane rectangular coordinate system. For the horizontal layered slope shown in Fig. 1, failure mechanism in the *m*th layer soil is located in a certain interval of logarithmic spiral equation. According to the law of slope failure, failure mechanism in the *m*th layer soil should satisfy two characteristics. Firstly, failure mechanism equation should be located in the fourth quadrant of plane Cartesian coordinate system. Secondly, failure mechanism equation should be strictly incremental function. Since failure mechanism equation is located in the fourth quadrant of planar rectangular coordinate system, *θ* ∈ [0, *π*/2]. The corresponding values of *x*_*m*_/*a*_*m*_ and *y*_*m*_/*a*_*m*_ can be calculated when *θ* = 0, 0.001, 0.002…*π*/2. According to the calculation results and the condition that am > 0, the increment interval of failure mechanism equation in the fourth quadrant can be determined. Least square method can be used to fit the function relation in the increment interval simultaneously, and the fitting result is *x* = *g*_*m*_(*a*_*m*_, *y*). For the horizontal layered slope, failure area of the *m*th layer soil can be expressed as

3 $$ \left\{ \begin{gathered} y_{n} + h_{n} + h_{n - 1} \cdots + h_{m + 1} \le y \le y_{n} + h_{n} + h_{n - 1} \cdots + h_{m} \hfill \\ f(y) \le x \le g_{m} (a_{m} {\kern 1pt} {\kern 1pt} {\kern 1pt} ,\;y) \hfill \\ \end{gathered} \right.. $$

### Establishment of virtual power equilibrium equation

Upper bound limit analysis analyzes slope stability from the angle of velocity and energy. Since energy is a scalar, it can effectively avoid disagreements of calculation results caused by different mapping methods. Upper bound limit analysis is based on virtual power equilibrium equation to calculate slope stability. That is to say, for any assumed slope failure mechanism, it indicates that slope is in a limit equilibrium state when external force power is equal to internal energy dissipation power. And slope stability can be analyzed and solved based on the limit equilibrium state. Therefore, for the slope failure mechanism established above, the calculation formulas for external force power and internal energy dissipation power of the slope failure area should be derived respectively.

#### External force power calculation

In natural state, external force power is all provided by gravity^37^. So for the *m*th layer soil of the horizontal layered slope, external force power *E*_*m*_ can be expressed as

4 $$ E_{m} = \int\limits_{{S_{m} }} {\gamma_{m} \cdot v \cdot {\text{cos}}\theta {\kern 1pt} dS} . $$

In Formula (4), *S*_*m*_ (m^2^) is failure area of the *m*th layer soil, *dS* (m^2^) is any area element in failure area of the *m*th layer soil, *γ*_*m*_ (kN/m^3^) is the density of corresponding area element, *v* (m/s) is the linear velocity of corresponding area element. Obviously, *v*·cos*θ* represents the gravity direction velocity of corresponding area element. According to the relation between linear velocity and angular velocity, gravity direction velocity of area element can be expressed *v*·cos*θ* = *ω*·*r*·cos*θ* = *ω*·*x*. Based on the gravity direction velocity calculation formula and the failure area expression, failure area external force power of the *m*th layer soil can be expressed as

5 $$ E_{m} = \omega \cdot \gamma_{m} \cdot \int\limits_{{S_{m} }} {xdS} = \omega \cdot \gamma_{m} \cdot \int_{f(y)}^{{g_{m} (a_{m} ,\,y)}} {\kern 1pt} \int_{{y_{n} + h_{n} + h_{n - 1} \cdots + h_{m + 1} }}^{{y_{n} + h_{n} + h_{n - 1} \cdots + h_{m} }} x dx. $$

Then external force power *E* of the slope failure area can be obtained by adding external force power of each soil layer, that is to say *E* = *ΣE*_*m*_.

#### Internal energy dissipation power calculation

In natural state, internal energy dissipation of the slope is concentrated on the velocity discontinuity surface. For horizontal layered slope, internal energy dissipation does not exist inside or outside the failure mechanism, but only occurs in the failure mechanism^38^. Selected *rdθ*/cos*φ*_*m*_ of failure mechanism in the *m*th layer soil as length element, and the cohesive force corresponding to the length element is *c*_*m*_*rdθ*/cos*φ*_*m*_. According to basic theory of plastic mechanics, friction energy dissipation and assumed dilatation energy dissipation can be completely offset, and internal energy dissipation is all provided by cohesion force^39^. Therefore, internal energy dissipation power *D*_*m*_ in the *m*th layer soil can be expressed as

6 $$ D_{m} = \int_{{\theta_{m} }}^{{\theta_{m + 1} }} {c_{m} \cdot v \cdot \cos \phi_{m} } \frac{rd\theta }{{\cos \phi_{m} }} = c_{m} \cdot \omega \cdot \int_{{\theta_{m} }}^{{\theta_{m + 1} }} {r^{2} } d\theta = \frac{{c_{m} \cdot \omega }}{{2\tan \phi_{m} }}(r_{m + 1}^{2} - r_{m}^{2} ). $$

In Formula (6), *r*_*m*_ (m) is line segment length connecting rotation center and the starting point of failure mechanism in the *m*th layer soil, *θ*_*m*_ (rad) is horizontal angle of the corresponding line segment, *r*_*m*+1_ (m) is line segment length connecting rotation center and the end point of failure mechanism in the *m*th layer soil, *θ*_*m*+1_ (rad) is horizontal angle of the corresponding line segment. According to the conversion relationship between polar coordinates and planar rectangular coordinates, *r*_*m*_ and *r*_*m*+1_ can be expressed as

7 $$ \left\{ \begin{gathered} r_{m} = \sqrt {g_{m}^{2} (a_{m} {\kern 1pt} {\kern 1pt} {\kern 1pt} ,\,y_{n} + h_{n} + h_{n - 1} \cdots + h_{m} ) + (y_{n} + h_{n} + h_{n - 1} \cdots + h_{m} )^{2} } \hfill \\ r_{m + 1} = \sqrt {g_{m + 1}^{2} (a_{m + 1} ,\,y_{n} + h_{n} + h_{n - 1} \cdots + h_{m + 1} ) + (y_{n} + h_{n} + h_{n - 1} \cdots + h_{m + 1} )^{2} } \hfill \\ \end{gathered} \right.. $$

According to Formula (6) and (7), internal energy dissipation power of the *m*th layer soil can be calculated. Then internal energy dissipation power *D* of the slope failure area can be obtained by adding internal energy dissipation power of each soil layer, that is to say *D* = *ΣD*_*m*_.

Thus, for the horizontal layered slope, both external force power and internal energy dissipation power in failure area can be calculated, and virtual power equilibrium equation can be established according to the condition that they are equal, and then the slope stability can be analyzed.

### Stability analysis and program development

Upper bound limit principle shows that external force power in failure area should be equal to internal energy dissipation power when the slope is in limit equilibrium state. For the slope that is not in equilibrium state, strength parameters of slope soil should be reduced repeatedly to make it gradually transition from non-limit equilibrium state to limit equilibrium state^40^. The reduction formula of slope soil strength is

8 $$ \left\{ \begin{gathered} c^{\prime} = \frac{c}{F} \hfill \\ \tan \phi^{\prime} = \frac{\tan \phi }{F} \hfill \\ \end{gathered} \right.. $$

In Formula (8), *c* and *φ* are cohesion and internal friction angle of primitive slope soil, while *c*′ and *φ*′ are cohesion and internal friction angle of slope soil after strength reduction. In the process of strength reduction, the least square fitting results will be affected because the internal friction angle needs to be reduced. Therefore, it is very complicated to reduce the limit equilibrium state manually. It is necessary to realize strength reduction of slope soil with help of computer programming cycle calculation.

In the process of circular program development, there are three problems to be solved. The first is initial conditions selection, the second is progressive relations establishment, and the last is termination conditions judgment. In view of the above three problems, this paper puts forward the following solutions. Firstly, for initial conditions selection, corresponding stability coefficient should be greater than 1.0 for a stable state slope. Therefore, reduction coefficient of 1.0 is selected as initial condition of the program. Secondly, for progressive relations establishment, reduction coefficient is gradually increased at intervals of 10^−1^. And combines with experience of slope engineering, the ratio function of external force power and internal energy dissipation power is established and defined as *ε* = (*D*/*E*)_min_. The slope state can be determined according to the range of *ε*. First of all, it indicates that slope is in a stable state and strength reduction coefficient is less than slope stability coefficient when *ε* is greater than 1. Secondly, it indicates that slope is in ultimate equilibrium state and strength reduction coefficient is equal to slope stability coefficient when *ε* is equal to 1. Lastly, it indicates that slope is in a failure state and strength reduction coefficient is greater than slope stability coefficient when *ε* is less than 1. And stability coefficient can be determined to be located in the range of length 10^−1^ according to the above two characteristics. Then reduction coefficient was gradually increased at intervals of 10^−2^ within the range. The process is repeated until stability coefficient meets the accuracy requirements. Lastly, for termination conditions judgment, the program can be terminated when the interval of slope stability coefficient can meet accuracy requirements. The program terminates at the interval of stability coefficient is located to be accurate to 10^−2^ according to practical needs of slope engineering.

## Analysis of engineering case

### Slope geological profile

**Figure 2:**
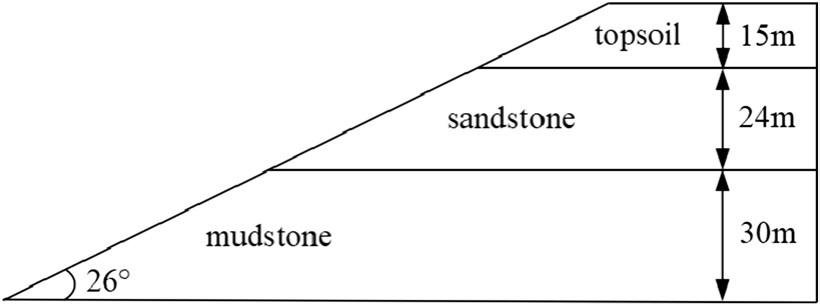
Slope morphology.

The mine excavation slope is selected as engineering background, and the slope angle is 26°. The slope is composed of topsoil, sandstone and mudstone from top to bottom. Among them, the thickness of topsoil is 15 m, the density of topsoil is 13.1 kN/m^3^, the cohesion of topsoil is 40 kPa, the internal friction angle of topsoil is 14.7°. The thickness of sandstone is 24 m, the density of sandstone is 19.3 kN/m^3^, the cohesion of sandstone is 75 kPa, the internal friction angle of sandstone is 16.9°. The thickness of mudstone is 30 m, the density of mudstone is 22.8 kN/m^3^, the cohesion of mudstone is 105 kPa, the internal friction angle of mudstone is 17.6°. The slope morphology is shown in Fig. 2.

### Slope stability analysis

The side slope equation can be expressed as *x* = *g*(*y*) = 2.05·*y* + *b* since the slope angle is 26°. Meanwhile, suppose the equation of slope bottom line is *y* = *y*_0_, the roof equation of mudstone or the bottom equation of sandstone can be expressed as *y* = *y*_0_ + 30. And the roof equation of sandstone or the bottom equation of topsoil can be expressed as *y* = *y*_0_ + 54. And the roof equation of topsoil can be expressed as *y* = *y*_0_ + 69. At the same time, according to the internal friction angle of topsoil is 14.7°, the logarithmic spiral equation of topsoil in polar coordinate can be expressed as

9 $$ r_{1} (\theta ) = a_{1} \cdot e^{0.262 \cdot \theta } . $$

By converting polar coordinates of the logarithmic spiral equation form of planar rectangular coordinates, it can be expressed as

10 $$ \left\{ \begin{gathered} \frac{x}{{a_{1} }} = e^{0.262 \cdot \theta } \cdot \cos \theta \hfill \\ \frac{y}{{a_{1} }} = e^{0.262 \cdot \theta } \cdot \sin \theta \hfill \\ \end{gathered} \right.. $$

**Figure 3:**
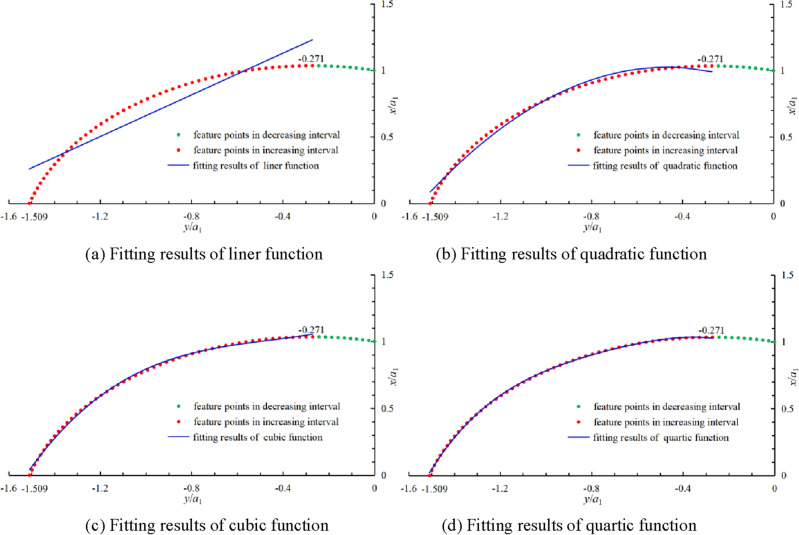
Least square fitting results.

Using discretization method previously introduced in this paper, the increment interval of the logarithmic spiral equation in the fourth quadrant is *y* ∈ [− 1.509*a*_1_, − 0.271*a*_1_]. The least squares fitting is performed on the logarithmic spiral equation in the increasing interval, the polynomial fitting image is shown in Fig. 3, and the fitting results and correlation coefficients are shown in Formula (11).

11 $$ \left\{ {\begin{array}{*{20}l} {\frac{x}{{a_{1} }} = 0.7922\frac{y}{{a_{1} }} + 1.4517} \hfill & {R^{2} = 0.8836} \hfill \\ {\frac{x}{{a_{1} }} = - \,0.8737\frac{{y^{2} }}{{a_{1}^{2} }} - 0.8279\frac{y}{{a_{1} }} + 0.8264} \hfill & {R^{2} = 0.9921} \hfill \\ {\frac{x}{{a_{1} }} = 0.672\frac{{y^{3} }}{{a_{1}^{3} }} + 0.97\frac{{y^{2} }}{{a_{1}^{2} }} + 0.6918\frac{y}{{a_{1} }} + 1.1873} \hfill & {R^{2} = 0.9985} \hfill \\ {\frac{x}{{a_{1} }} = - \,0.9209\frac{{y^{4} }}{{a_{1}^{4} }} - 2.6704\frac{{y^{3} }}{{a_{1}^{3} }} - 3.2635\frac{{y^{2} }}{{a_{1}^{2} }} - 1.4829\frac{y}{{a_{1} }} + 0.8133} \hfill & {R^{2} = 0.9997} \hfill \\ \end{array} } \right.. $$

According to formula (11), when quartic function is used for polynomial fitting, its correlation coefficient *R*^2^ = 0.9997 can fully meet the requirements of the program. Therefore, the fitting result of topsoil failure mechanism can be expressed as

12 $$ g_{1} (a_{1} ,{\kern 1pt} y) = - 0.9209y^{4} /a_{1}^{3} - 2.6704y^{3} /a_{1}^{2} - 3.2635y^{2} /a_{1} - 1.4829y + 0.8133a_{1} . $$

Similarly, the increment interval of the logarithmic spiral equation in sandstone in the fourth quadrant is *y* ∈ [− 1.612*a*_2_, − 0.318*a*_2_]. And the fitting result of sandstone failure mechanism can be expressed as

13 $$ g_{2} (a_{2} ,{\kern 1pt} {\kern 1pt} y) = - \,0.6581y^{4} /a_{2}^{3} - 2.0429y^{3} /a_{2}^{2} - 2.7943y^{2} /a_{2} - 1.382y + 0.8186a_{2} . $$

The increment interval of the logarithmic spiral equation in mudstone in the fourth quadrant is *y* ∈ [− 1.645*a*_3_, − 0.333*a*_3_]. And the fitting result of mudstone failure mechanism can be expressed as

14 $$ g_{3} (a_{3} {\kern 1pt} ,y) = - \,0.6023y^{4} /a_{3}^{3} - 1.9207y^{3} /a_{3}^{2} - 2.6711y^{2} /a_{3} - 1.3937y + 0.8111a_{3} . $$

Therefore, external force power of the slope failure area can be expressed as

15 $$ E = \omega \cdot \left( {13.1 \cdot \int_{{y_{0} + 54}}^{{y_{0} + 69}} x dx + 19.3 \cdot \int_{f(y)}^{{g_{2} (a_{2} ,y)}} {\int_{{y_{0} + 30}}^{{y_{0} + 54}} x dx + 22.8 \cdot } \int_{f(y)}^{{g_{3} (a_{3} ,y)}} {\int_{{y_{0} }}^{{y_{0} + 30}} x dx} } \right). $$

On the other side, internal energy dissipation power of the slope failure area can be expressed as

16 $$ \begin{aligned} D = & \omega \cdot \{ 76.24 \cdot [g_{1}^{2} (a_{1} {\kern 1pt} {\kern 1pt} {\kern 1pt} ,y_{0} + 54) + (y_{0} + 54)^{2} - g_{1}^{2} (a_{1} {\kern 1pt} {\kern 1pt} {\kern 1pt} ,y_{0} + 69) - (y_{0} + 69)^{2} ] \\ & + 123.43 \cdot [g_{2}^{2} (a_{2} {\kern 1pt} {\kern 1pt} {\kern 1pt} ,y_{0} + 30) + (y_{0} + 30)^{2} - g_{2}^{2} (a_{2} {\kern 1pt} {\kern 1pt} {\kern 1pt} ,y_{0} + 54) - (y_{0} + 54)^{2} ] \\ & + 165.5 \cdot [g_{3}^{2} (a_{3} {\kern 1pt} {\kern 1pt} {\kern 1pt} ,y_{0} ) + y_{0}^{2} - g_{3}^{2} (a_{3} {\kern 1pt} {\kern 1pt} {\kern 1pt} ,y_{0} + 30) - (y_{0} + 30)^{2} ]\} . \\ \end{aligned} $$

In the process of solving ratio function *ε*, the following three constraints should be satisfied according to the experience of slope engineering.

First of all, the failure mechanism should be located in increasing interval of the fourth quadrant. This condition can be expressed as

17 $$ \left\{ {\begin{array}{*{20}l} { - \,1.509 \cdot a_{1} \le y_{0} + 69 \le - \,0.271 \cdot a_{1} } \hfill & { - \,1.509 \cdot a_{1} \le y_{0} + 54 \le - \,0.271 \cdot a_{1} } \hfill \\ { - \,1.612 \cdot a_{2} \le y_{0} + 54 \le - \,0.318 \cdot a_{2} } \hfill & { - \,1.612 \cdot a_{2} \le y_{0} + 30 \le - \,0.318 \cdot a_{2} } \hfill \\ { - \,1.645 \cdot a_{3} \le y_{0} + 30 \le - \,0.333 \cdot a_{3} } \hfill & { - \,1.645 \cdot a_{3} \le y_{0} \le - \,0.333 \cdot a_{3} } \hfill \\ \end{array} } \right.. $$

What is more, the failure mechanism should be located inside slope body. That is to say, for any ordinate, the corresponding abscissa of failure mechanism should be larger than that of slope sliding surface. This condition can be expressed as

18 $$ \left\{ {\begin{array}{*{20}l} {g_{1} (a_{1} {\kern 1pt} {\kern 1pt} {\kern 1pt} ,y) - f(y) \ge 0} \hfill & {y \in [y_{0} + 54{\kern 1pt} {\kern 1pt} {\kern 1pt} ,y_{0} + 69]} \hfill \\ {g_{2} (a_{2} {\kern 1pt} {\kern 1pt} {\kern 1pt} ,y) - f(y) \ge 0} \hfill & {y \in [y_{0} + 30{\kern 1pt} {\kern 1pt} ,y_{0} + 54]} \hfill \\ {g_{3} (a_{3} {\kern 1pt} {\kern 1pt} {\kern 1pt} ,y) - f(y) \ge 0} \hfill & {y \in [y_{0} {\kern 1pt} ,y_{0} + 30]} \hfill \\ \end{array} } \right.. $$

Finally, the coordinates of toe point and failure mechanisms on the soil interfaces are consistent. This condition can be expressed as

19 $$ \left\{ \begin{gathered} g_{1} (a_{1} {\kern 1pt} {\kern 1pt} {\kern 1pt} ,y{}_{0} + 54) = g_{2} (a_{2} {\kern 1pt} {\kern 1pt} {\kern 1pt} ,y{}_{0} + 54){\kern 1pt} \hfill \\ g_{2} (a_{2} {\kern 1pt} {\kern 1pt} {\kern 1pt} ,y{}_{0} + 30) = g_{3} (a_{3} {\kern 1pt} {\kern 1pt} {\kern 1pt} ,y{}_{0} + 30){\kern 1pt} \hfill \\ g_{3} (a_{3} {\kern 1pt} {\kern 1pt} {\kern 1pt} ,y{}_{0}) = f(y{}_{0}) \hfill \\ \end{gathered} \right.. $$

**Figure 4:**
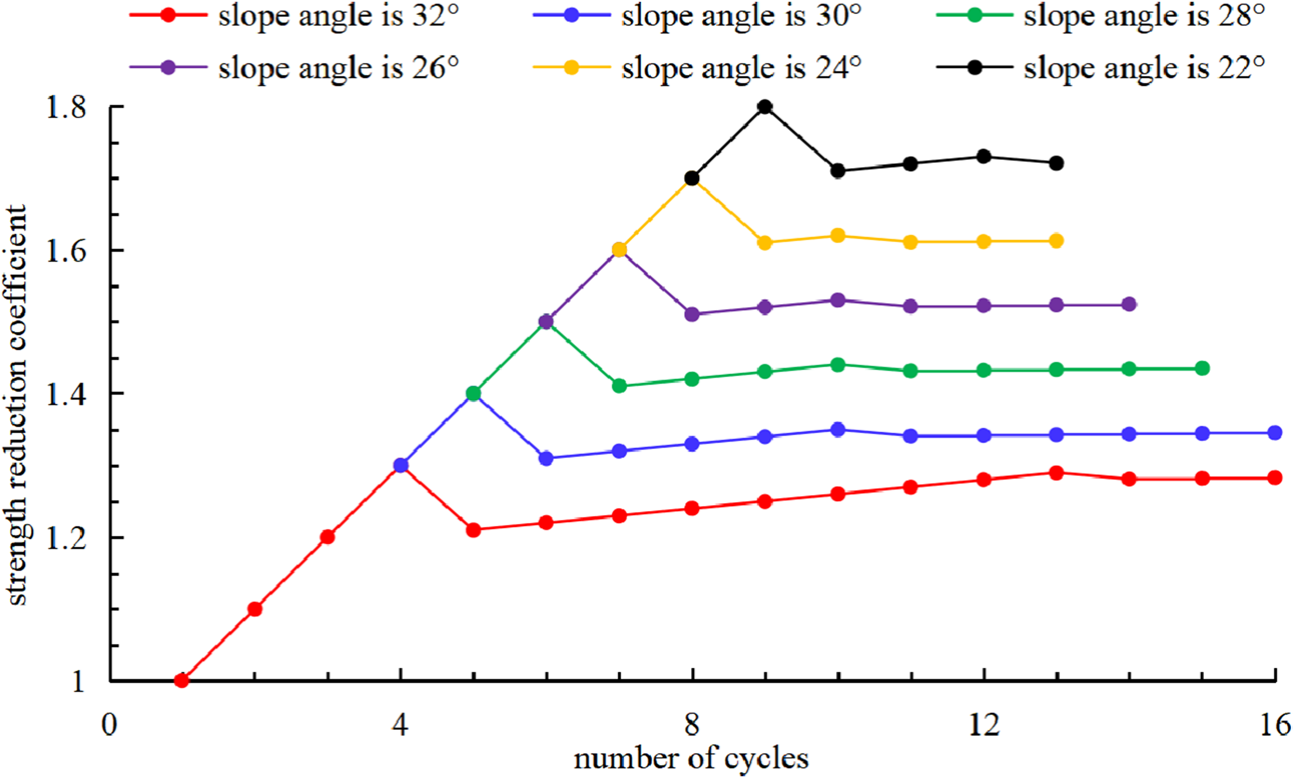
Processes of strength reduction.

According to calculation formulas of external force power and internal energy dissipation power, and combines with the above three constraints, ratio function *ε* corresponding to strength reduction coefficient of 1.0 can be solved. When strength reduction coefficient is 1.0, the corresponding ratio function *ε* is greater than 1, indicating that slope is in a stable state. Then strength reduction coefficient is gradually increased at intervals of 10^−1^, and the above calculation process is repeated. Finally, when strength reduction coefficient is 1.6, the corresponding ratio function *ε* is less than 1, indicating that slope is in a failure state. Slope stability coefficient is between 1.5 and 1.6 according to the above analysis. After that, slope stability coefficient can be obtained between 1.52 and 1.53 by repeating the above calculation process with intervals of 10^−2^. And finally, slope stability coefficient can be obtained between 1.523 and 1.524 by repeating the above calculation process with intervals of 10^−3^. Therefore, corresponding stability coefficient is 1.52 when slope angle is 26°. In order to research the influence of slope angle on stability coefficients, using the same method calculates stability coefficients corresponding to slope angles of 22°, 24°, 28°, 30° and 32°. The processes of strength reduction corresponding to different slope angles are shown in Fig. 4.

According to Fig. 4, when slope angle is 22°, 24°, 26°, 28°, 30° and 32°, the corresponding strength reduction coefficient converges to 1.72, 1.61, 1.52, 1.43, 1.35 and 1.28 through 12–16 times of cycle calculation, and slope stability coefficient is equal to the corresponding strength reduction coefficient. It indicates that slope stability coefficient can be calculated through less than 20 times of cycle calculation for different slope angles, which makes the calculation process simpler and faster compared with traditional calculation process. Meanwhile, slope stability coefficient increases and the increasing rate accelerates gradually with the decreasing of slope angle. Therefore, slope stability coefficient can be improved by reducing slope angle in engineering practice.

### Calculation result evaluation

**Figure 5:**
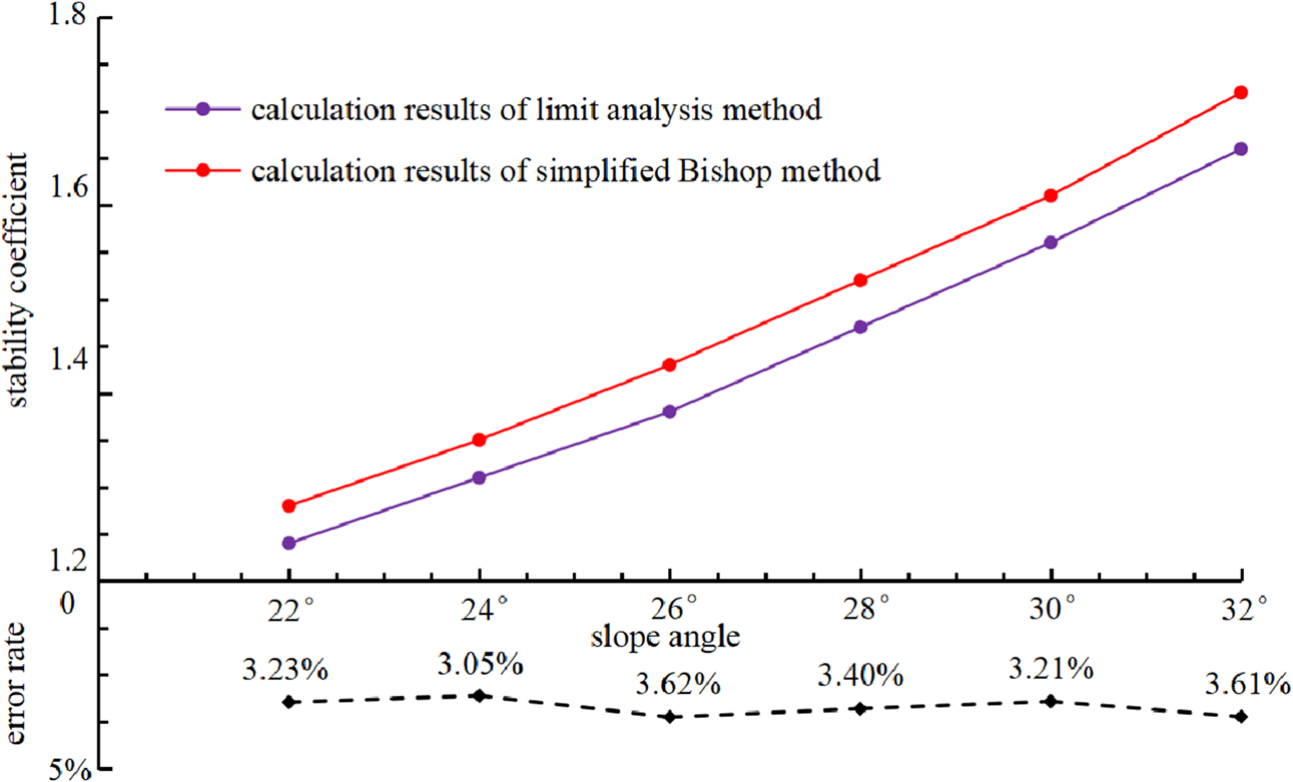
Calculation results of stability coefficient and error rate.

In this paper, simplified Bishop method is used to evaluate accuracy of calculation results. Simplified Bishop method assumes the sliding surface is circular and ignores the inter-slice shear forces, and calculates stability coefficient by establishing static equilibrium equation and torque equilibrium equation. Simplified Bishop method and limit analysis method are used to analyze slope stability from different angles, but their results should be highly consistent. When slope angle are 22°, 24°, 26°, 28°, 30° and 32°, calculation results of stability coefficient and error rate of two methods are shown in Fig. 5.

It can be seen from Fig. 5, error rate of stability coefficient calculation results of two methods is less than 5% for different slope angles, which can meet requirements of engineering practice. The calculation error is convenient to estimate and eliminate due to stability coefficient calculation results of upper bound limit analysis are strict upper bound solutions. Meanwhile, upper bound limit analysis method analyzes slope stability through virtual power equilibrium equations, and can avoid calculation error caused by mapping inconsistency because of virtual power is a kind of scalar quantity^41^. To sum up, upper bound limit analysis method has certain applicability in slope engineering practice.

## Conclusion

Based on upper bound limit principle and strength reduction principle, this paper proposed a stability analysis method suitable for horizontal stratified slope, and evaluates accuracy of calculation results combined with engineering practice. The main conclusions are as follows.

1. Based on associated flow law and velocity separation requirements, the failure mechanism of horizontal layered slope was established. The calculation method of external force power and internal energy dissipation power of slope was proposed by using the least square fitting method, and virtual power equilibrium equation of horizontal layered slope was formed.
2. The cyclic flow of slope stability analysis was put forward based on the strength reduction principle, and the horizontal stratified slope stability analysis system was developed combining with computer programming technology.
3. Taking typical mine excavation slope as engineering background, accuracy of calculation results of the stability analysis system was evaluated by combining simplified Bishop method. The error rate of stability coefficient of two methods was between 3 and 5%, which can meet the requirements of engineering practice.

## Data Availability

The data used to support the findings of this study are available from the corresponding author upon request.
